# General features, management strategies, and outcomes of symptomatic spontaneous isolated celiac artery dissection

**DOI:** 10.3389/fsurg.2022.972276

**Published:** 2022-10-21

**Authors:** Fushuo Zhou, Zhi Zheng, Youmin Pan

**Affiliations:** Division of Cardiothoracic and Vascular Surgery, Tongji Hospital, Tongji Medical College, Huazhong University of Science and Technology, Wuhan, China

**Keywords:** celiac artery dissection, conservative treatment, endovascular treatment, abdominal pain, outcome

## Abstract

**Objectives:**

Spontaneous isolated celiac artery dissection (SICAD) is a rare condition that has not been fully investigated and reported, and very little is known regarding its prognosis and management. Here, we aimed to provide more evidence on the management strategy and outcome for symptomatic SICAD based on the experience of a single center.

**Methods:**

From January 2018 to December 2021, a total of consecutive 51 patients with symptomatic SICAD were retrospectively included in this study. These patients had been selectively treated with conservative treatment (*n* = 31) or endovascular treatment (*n* = 20). Baseline data, imaging findings, treatment strategy, outcomes, and follow-up data have been described and analyzed.

**Results:**

The mean age of the patients was 53.2 ± 9.6 years, 44 (86.3%) were male, and 36 (70.6%) had hypertension. The median length of stay was 10.0 days. The complete remission rate was 92.2% on discharge. The median follow-up time was 21.0 months. A secondary intervention was required for two patients during follow-up in the conservative group, wherein one underwent a stent placement three months after discharge because of progression of symptoms and extension of dissection, and the other required intervention one month after discharge because of symptomatic progression. No secondary intervention was required in the endovascular group. Occasional and mild relapse of symptoms occurred in two patients in both the conservative and endovascular groups, with no secondary intervention. The length of dissection (25.5 ± 11.8 mm vs. 19.1 ± 7.4 mm, *P* = 0.022) and complete remodeling rate (85.7% vs. 15.4%, *P* < 0.001) in the endovascular group were greater than that in the conservative group.

**Conclusion:**

Patients with symptomatic SICAD who were selectively treated with conservative treatment or endovascular treatment had satisfactory early and medium-term outcomes. Endovascular treatment showed significant advantages in the complete remodeling of the celiac artery and presented with a lower rate of secondary intervention. Moreover, it was found to be a safe and effective remedy for failed conservative treatment.

## Introduction

Spontaneous isolated celiac artery dissection (SICAD), defined as the dissection of the celiac artery without aortic dissection, is a rare condition (1, 2). The clinical manifestation of SICAD is non-specific, and there is a certain misdiagnosis rate (3). With the increased evaluation of abdominal pain by computed tomography angiography (CTA), SICAD has been diagnosed more frequently in recent years (4, 5). However, to the best of our knowledge, just over three hundred cases have been reported in previous literature (2, 3, 5–9). Although some cases without symptoms were found accidentally, some patients suffered from refractory symptoms and were even under the potential risk of organ ischemia, aneurysmal dilatation, and arterial rupture (6, 10–15). Owing to small sample sizes and different treatment algorithms used in various centers, the natural prognosis of SICAD remains uncertain, and there is no consensus yet on standardized management strategies for the lesion (2, 6, 10, 11, 14, 16–18).

In the current study, we report the experience of a single center with the largest sample size to provide more evidence on the management strategy and outcome for symptomatic SICAD.

## Materials and methods

### Study design and collection of cases

From January 2018 to December 2021, a total of 51 consecutive patients with symptomatic SICAD diagnosed by CTA in our center were retrospectively analyzed. Patients without symptoms or a CTA scan on admission were excluded before the initial data analysis. Symptomatic SICAD was defined as having one or more symptoms after the onset of SICAD, excluding other causes. The demographics, comorbidities, symptoms, laboratory data, imaging findings, treatment strategy, outcomes, and follow-up data were recorded and analyzed. Imaging findings included the length of dissection, the maximum diameter of dissection, the minimum diameter of the true lumen, the diameter of the normal celiac artery, the extent of lesion involvement, dissecting aneurysm, and morphological classification. The dissecting aneurysm was defined as a diameter more than 1.5 times the diameter of the normal celiac artery adjacent to dissection (19). The morphological classification was described based on the types proposed by Kim SR et al. (11): type IA only had the dissection flap, type IB had the dissection flap accompanied by false lumen thrombosis, type IIA only had the intramural hematoma, and type IIB had the intramural hematoma accompanied by penetrating ulcer or focal dissection flap.

### Treatment strategy selection

The recommendation for conservative treatment alone or endovascular treatment was provided mainly based on the clinical characteristics and imaging findings of each patient reviewed by the treating specialist. In general, endovascular treatment was recommended for patients with severe persistent symptoms or relapse of symptoms despite conservative treatment, as well as if severe organ ischemia, an extension of dissection, rupture, and impending rupture was suspected. The severe persistent symptom was defined as progression or no remission of symptoms. The relapse of symptoms was defined as the relapse of symptoms after remission. Written informed consent was obtained from the patient and the next of kin for endovascular treatment. Other patients were recommended for conservative treatment alone. All patients were treated with basic medical treatment at the specialists’ discretion case-by-case. Methods for conservative treatment included fasting, parenteral nutrition, blood pressure control, antiplatelet therapy, anticoagulant therapy, and analgesic therapy.

### Follow-up schedule

Follow-up was carried out by outpatient visits, inpatient visits, and telephone calls. We mainly focused on the relapse or progression of symptoms, secondary intervention, and imaging changes. The complete disappearance of the dissection was an indication of a complete remodeling of the celiac artery. Follow-up was terminated in May 2022 or if the relapse of symptoms, progression of symptoms, secondary intervention, and death occurred.

### Statistical analysis

Categorical variables, expressed as frequency and percentages, were compared by Pearson’s *χ*^2^ test or Fisher's exact test. Continuous variables, expressed as mean ± standard deviation or median (first quartile, third quartile), were compared using the independent samples *t*-test or Mann–Whitney U test. *P*-value was tested on two sides. *P* < 0.05 was considered to be statistically significantly different. SPSS software (version 26, IBM, Armonk, NY, USA) was used for statistical analysis.

## Results

### Baseline data

A total of 51 patients with symptomatic SICAD were included in the analysis. Of these, 31 were treated with conservative treatment and 20 with endovascular treatment. The mean age was 53.2 ± 9.6 years (range, 32 to 77 years), 44 (86.3%) were male, and the mean body mass index (BMI) was 25.0 ± 3.4 kg/m^2^ (range, 16.6 to 34.0 kg/m^2^). The median prehospital time was 4.0 (1.0, 11.0) days. Seventeen (33.3%) and eleven (21.6%) patients were smokers and drinkers, respectively. Further, 36 (70.6%) had hypertension, 6 (11.8%) had diabetes mellitus, 21 (41.2%) had hyperlipidemia, 9 (17.6%) had hyperuricemia, 17 (33.3%) had aortic calcification, and 12 (23.5%) had a history of abdominal operation. Forty-two (82.4%) had abdominal pain, eight (15.7%) had chest pain, seven (13.7%) had back pain, and two (3.9%) had lower back pain. Other symptoms consisted of nausea and vomiting (9/51, 17.6%), abdominal distention (6/51, 11.8%), and hematochezia (2/51, 3.9%). The laboratory results were unremarkable. Among these baseline data, the BMI in the endovascular group was larger than that in the conservative group (26.3 ± 2.8 kg/m^2^ vs. 24.1 ± 3.5 kg/m^2^, *P* = 0.022), while all other baseline characteristics showed no significant differences between the two groups (*P* > 0.05). The baseline data are summarized in Table 1.

**Table 1:** Baseline data and CTA findings of the patients with symptomatic SICAD.

	Total (*n* = 51)	Conservative treatment (*n* = 31)	Endovascular treatment (*n* = 20)	*P*-value
Male, *n* (%)	44 (86.3)	26 (83.9)	18 (90.0)	0.690
Age, years	53.2 ± 9.6	53.0 ± 10.0	53.5 ± 9.1	0.867
BMI, kg/m^2^	25.0 ± 3.4	24.1 ± 3.5	26.3 ± 2.8	0.022
Prehospital time, days	4.0 (1.0,11.0)	3.0 (1.0,15.0)	4.5 (1.0,8.8)	0.874
Smoking, *n* (%)	17 (33.3)	8 (25.8)	9 (45.0)	0.156
Drinking, *n* (%)	11 (21.6)	5 (16.1)	6 (30.0)	0.304
**Comorbidities, *n* (%)**				
Hypertension	36 (70.6)	19 (61.3)	17 (85.0)	0.070
Diabetes mellitus	6 (11.8)	3 (9.7)	3 (15.0)	0.668
Hyperlipidemia	21 (41.2)	14 (45.2)	7 (35.0)	0.472
Hyperuricemia	9 (17.6)	3 (9.7)	6 (30.0)	0.129
Aortic calcification	17 (33.3)	8 (25.8)	9 (45.0)	0.156
History of abdominal operation	12 (23.5)	7 (22.6)	5 (25.0)	1.000
**Symptoms, *n* (%)**				
Abdominal pain	42 (82.4)	27 (87.1)	15 (75.0)	0.289
Chest pain	8 (15.7)	4 (12.9)	4 (20.0)	0.696
Back pain	7 (13.7)	3 (9.7)	4 (20.0)	0.411
Lower back pain	2 (3.9)	1 (3.2)	1 (5.0)	1.000
Nausea and vomiting	9 (17.6)	6 (19.4)	3 (15.0)	1.000
Abdominal distention	6 (11.8)	3 (9.7)	3 (15.0)	0.668
Hematochezia	2 (3.9)	1 (3.2)	1 (5.0)	1.000
**Laboratory examination**				
Alanine transaminase, U/L	18.0 (13.0, 30.0)	18.0 (14.0,26.0)	18.0 (13.0,32.3)	0.809
Total bilirubin, μmol/L	12.2 (9.2,18.9)	11.1 (9.0,21.7)	13.0 (10.6,18.3)	0.499
Serum creatinine, μmol/L	75.7 ± 14.4	76.7 ± 14.0	74.2 ± 15.3	0.544
White blood cell, 10^9^/L	7.6 (5.7,9.8)	7.6 (5.7,9.8)	7.6 (5.2,10.3)	0.885
Neutrophilic granulocyte, %	70.7 ± 10.0	70.8 ± 11.2	70.5 ± 8.1	0.900
Platelet count, 10^12^/L	214.2 ± 87.2	214.6 ± 94.7	213.6 ± 76.4	0.967
**Imaging findings**				
Length of dissection, mm	21.6 ± 9.8	19.1 ± 7.4	25.5 ± 11.8	0.022
Diameter of CA at the ostium, mm	8.2 ± 1.6	8.0 ± 1.6	8.4 ± 1.7	0.452
Diameter of CA adjacent to dissection, mm	7.5 ± 1.4	7.5 ± 1.4	7.6 ± 1.3	0.807
Maximum diameter of dissection, mm	12.0 ± 4.4	11.2 ± 2.1	13.3 ± 6.4	0.101
Minimum diameter of true lumen, mm	3.3 ± 1.6	3.4 ± 1.7	3.1 ± 1.4	0.517
Dissecting aneurysm, *n* (%)	26 (51.0)	16 (51.6)	10 (50.0)	0.910
** Extent of lesion involvement, *n* (%)**				
Only CA	22 (43.1)	11 (35.5)	11 (55.0)	0.169
CA and CHA	2 (3.9)	1 (3.2)	1 (5.0)	1.000
CA and SA	4 (7.8)	4 (12.9)	0 (0)	0.145
CA and LGA	2 (3.9)	2 (6.5)	0 (0)	0.514
CA and SA and CHA	16 (31.4)	10 (32.3)	6 (30.0)	0.865
CA and SA and LGA	1 (2.0)	0 (0)	1 (5.0)	0.392
CA and SA and CHA and LGA	4 (7.8)	3 (9.7)	1 (5.0)	1.000
** Classification, *n* (%)**				
IA	27 (52.9)	17 (54.8)	10 (50.0)	0.735
IB	17 (33.3)	8 (25.8)	9 (45.0)	0.156
IIA	5 (9.8)	4 (12.9)	1 (5.0)	0.636
IIB	2 (3.9)	2 (6.5)	0	0.514

CTA, computed tomography angiography; SICAD, spontaneous isolated celiac artery dissection; BMI, body mass index; CA, celiac artery; CHA, common hepatic artery; SA, splenic artery; LGA, left gastric artery.

### CTA findings

All 51 patients with symptomatic SICAD had available CTA scans upon admission. The mean length of dissection was 21.6 ± 9.8 mm, the mean diameter of the celiac artery at the ostium was 8.2 ± 1.6 mm, the diameter of the celiac artery adjacent to dissection was 7.5 ± 1.4 mm, the maximum diameter of dissection was 12.0 ± 4.4 mm, and the minimum diameter of the true lumen was 3.3 ± 1.6 mm. The dissecting aneurysm was found in 26 (51.0%) patients. The extent of lesion involvement in 22 (43.1%) patients was only limited to the celiac artery, followed by the simultaneous involvement of the celiac artery, splenic artery, and the common hepatic artery (*n* = 16, 31.4%). In addition, no occlusion of the branches was observed. Type IA was found in 27 (52.9%), type IB in 17 (33.3%), type IIA in 5 (9.8%), and type IIB in 2 (3.9%) patients. Among these CTA findings, the length of dissection was larger in the endovascular group than that in the conservative group (25.5 ± 11.8 mm vs. 19.1 ± 7.4 mm, *P* = 0.022), and there were no statistically significant differences in any of the other findings between the two groups (*P* > 0.05). The imaging findings are shown in Table 1.

### Treatment and outcomes

In the cohort, 13 (25.5%) patients showed some degree of fasting, 44 (86.3%) patients used antihypertensive agents, and the proportion of the use of antiplatelet therapy alone, anticoagulant therapy alone, and both was 9.8% (5/51), 25.5% (13/51), and 11.8% (6/51), respectively. All patients had a certain symptomatic remission on discharge. The complete remission rate was 92.2% (47/51), with the conservative group and the endovascular group showing rates of 90.3% (28/31) and 95.0% (19/20), respectively. The median length of stay was 10.0 (5.0, 13.0) days. In addition, three (5.9%) patients were initially misdiagnosed, with pancreatitis in two cases and gastritis in one case. In the endovascular group, general anesthesia was used in 14 (70.0%) and local anesthesia in 6 (30.0%) patients. Nineteen (95.0%) of the patients were approached from the femoral artery access and only one (5.0%) was approached from the brachial artery. Nineteen of the patients had 21 stents (17 covered stents and 4 bare stents) deployed into the celiac artery. One had adjunctive coils embolization of the false lumen of the splenic artery, and two had adjunctive balloon angioplasty to achieve a more ideal effect after stent placement. All stents deployed into the celiac artery were self-expandable. The diameters of the selected stents ranged from 6 mm to 8 mm, and the lengths were 25 mm to 60 mm. For one remaining patient, the dissection involved the narrow ostium of the celiac artery and was accompanied by a giant pseudoaneurysm and perivascular hematoma that prevented the placement of the stents and compressed the abdominal aorta, superior mesenteric artery, and the left renal artery. A covered stent graft was deployed in the abdominal aorta above the ostium of the superior mesenteric artery for occluding the ostium of the celiac artery to rapidly reduce the blood flow through the false lumen preventing an imminent rupture. The blood flow in the false lumen nearly disappeared after the stent was deployed, without malperfusion occurring in the downstream organs. Procedure-related complications occurred in two patients. One had bleeding of the femoral artery and underwent an incision in the puncture site for hemostasis. The other one had a focal dissection in the right external iliac artery and only follow-up was recommended. The treatment and outcomes are shown in Table 2.

**Table 2:** Treatments and outcomes of the patients with symptomatic SICAD in hospital.

	Total (*n* = 51)	Conservative treatment (*n* = 31)	Endovascular treatment (*n* = 20)	*P*-value
**Medical therapy, *n* (%)**				
Blood pressure control	44 (86.3)	25 (80.6)	19 (95.0)	0.223
Antiplatelet alone	5 (9.8)	2 (6.5)	3 (15.0)	0.369
Anticoagulant alone	13 (25.5)	7 (22.6)	6 (30.0)	0.553
Antiplatelet and anticoagulant	6 (11.8)	0	6 (30.0)	0.002
**Endovascular procedures, *n* (%)**				
Stent placement	/	/	19 (95.0)	/
Coil embolization	/	/	1 (5.0)	/
Balloon angioplasty	/	/	2 (10.0)	/
Coverage of celiac artery	/	/	1 (5.0)	/
**Access approach, *n* (%)**				
Femoral artery	/	/	19 (95.0)	/
Brachial artery	/	/	1 (5.0)	/
**Anesthesia, *n* (%)**				
General anesthesia	/	/	14 (70.0)	/
Local anesthesia	/	/	6 (30.0)	/
Operation time, minutes	/	/	122.5 (82.0, 158.3)	/
**Outcomes, *n* (%) or days**				
Initial misdiagnosis	3 (5.9)	1 (3.2)	2 (10.0)	0.553
Length of stay	10.0 (5.0,13.0)	7.0 (5.0, 12.0)	11.5 (9.0, 14.0)	0.018
Complete remission	47 (92.2)	28 (90.3)	19 (95.0)	1.000
Partial remission	4 (7.8)	3 (9.7)	1 (5.0)	1.000
Complication	2 (3.9)	0	2 (10.0)^a^	0.149

SICAD: spontaneous isolated celiac artery dissection.

^a^ One patient had bleeding in the puncture site of the right femoral artery and had a simultaneous incision and hemostasis, and another patient had a local dissection in the right external iliac artery and was suggested follow-up only.

### Follow-up

**Figure 1:**
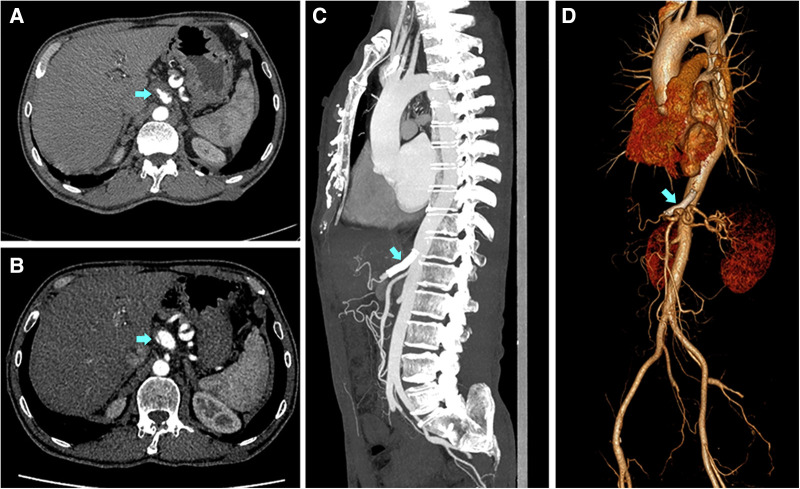
Imaging findings of the therapeutic process for one patient for whom the conservative treatment failed. A 51-year-old Male with a complaint of abdominal pain for 1 day was diagnosed with symptomatic spontaneous isolated celiac artery dissection. (**A**) The imaging presented intramural hematoma accompanied by a penetrating ulcer. The patient was subjected to conservative treatment. (**B**) Three months after discharge, the patient had a symptomatic progression. The imaging showed the lesion had a severe extension with a visible dissection flap and narrow true lumen 3 months after discharge. The patient then underwent stent placement. (**C,D**) The stent was patent and the celiac artery had a complete remodeling with false lumen disappearance and true lumen recovery at the one-year follow-up after the secondary intervention.

**Figure 2:**
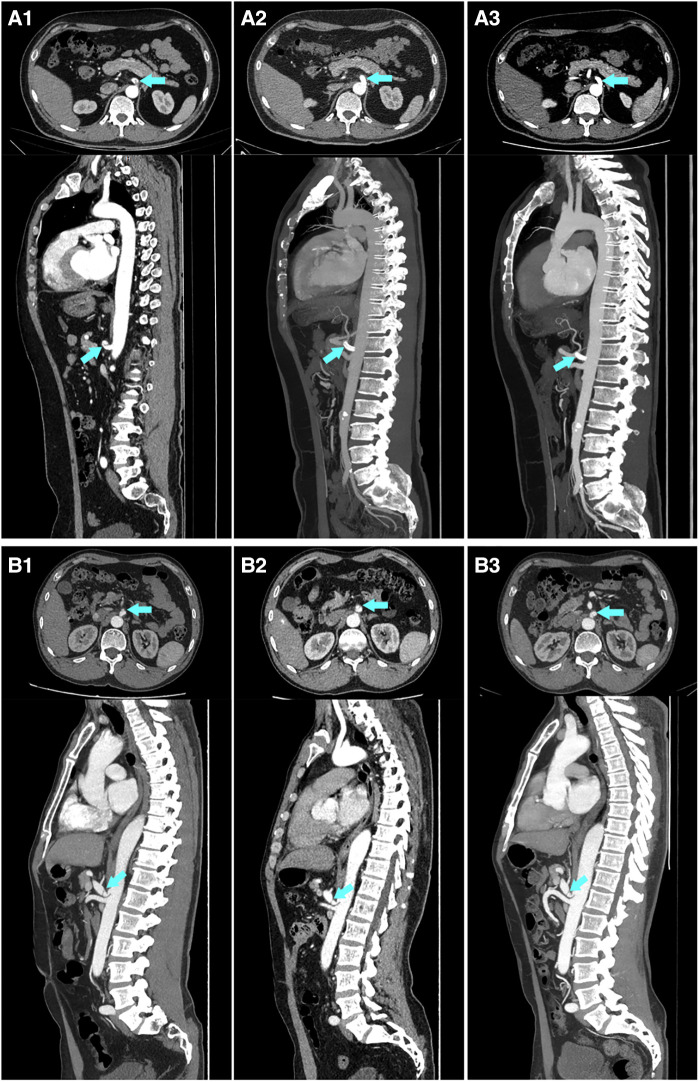
An example of a comparison of imaging follow-up for the conservative treatment and endovascular treatment groups. (**A**) A 46-year-old Male, who was diagnosed with symptomatic spontaneous isolated celiac artery dissection, underwent endovascular treatment. A1–A3 are the imaging scans before stent implantation, one week after stent implantation, and 12 months after stent implantation, respectively, showing a complete and stable remodeling of the celiac artery within a short term. (**B**) A 59-year-old Male, who was diagnosed with symptomatic spontaneous isolated celiac artery dissection, underwent conservative treatment. B1–B3 are the imaging scans presenting a persistent patent false lumen on admission, at follow-up of 3 months and 39 months, respectively.

Six patients in the cohort were not available for follow-up once discharged. The median follow-up time was 21.0 (12.5, 39.0) months. A secondary intervention was required in two patients (7.7%, 2/26) in the conservative group. One underwent a stent placement three months after discharge because of severe extension of dissection and progression of symptoms (Figure 1), and the other one occurred one month after discharge because of the progression of symptoms alongside severe psychological burden. The problems in both patients were effectively solved after the endovascular intervention. No secondary endovascular intervention was required in the endovascular group. Occasional and mild relapse of symptoms occurred in two patients in both the conservative and endovascular groups (7.7%, 2/26 vs. 10.5%, 2/19). Dual antiplatelet therapy was reactivated in one due to a focal opacification in the stent while a regular outpatient visit was recommended for the remaining. Furthermore, 13 patients in the conservative treatment group and 14 in the endovascular treatment group had at least one subsequent CTA scan after the corresponding treatment. In the conservative group, apart from the aforementioned one who underwent secondary intervention due to an extension of dissection, one had a new small intramural hematoma distal to the celiac artery at the third-year follow-up. A regular outpatient follow-up was recommended for the patient as the total extent of the dissection showed a decreasing trend and there were no symptoms. In the endovascular group, all the employed stents had good patency without stenosis. In addition to the aforementioned one who had a focal opacification in the stent, one had partial thrombosis in the common hepatic artery downstream of the stent but no symptoms; antiplatelet therapy was recommended for the patient. Favorable imaging changes or no progression were noted in the available CTA of the remaining patients. The complete remodeling rate of the celiac artery in the endovascular group was significantly higher than that in the conservative group (85.7%, 12/14 vs. 15.4%, 2/13; *P* < 0.001). An example of a comparison of the remodeling process for both groups is shown in Figure 2. Additionally, we recorded the follow-up results based on the morphological classification in the conservative group. Symptomatic relapse occurred in one case of type IA (1/13, 7.7%) and one case of type IIB (1/2, 50.0%), while neither type IB nor type IIA cases showed symptomatic relapse. One patient with type IA underwent secondary intervention (1/13, 7.7%), and one patient with type IIB underwent secondary intervention (1/2, 50.0%); however, no patient with type IB or type IIA underwent secondary intervention. Furthermore, from the available CTA scans, complete remodeling was found in one case (1/2, 50.0%) of type IB and one case of type IIA (1/3, 33.3%), while none of the patients with type IA or type IIB achieved complete remodeling. The main follow-up data of 45 patients are summarized in Table 3.

**Table 3:** Follow-up data of the patients with symptomatic SICAD.

Number	Treatment	Relapse or progression of symptoms (time)	Secondary intervention (time)	Complete remodeling	CTA scan timing	Number	Treatment	Relapse or progression of symptoms (time)	Secondary intervention (time)	Complete remodeling	CTA scan timing
1	Conservative	No	No	/	/	15	Endovascular	Yes (32 month)^a^	No	/	/
2	Conservative	No	No	/	/	21	Endovascular	No	No	/	/
3	Conservative	No	No	/	/	22	Endovascular	Yes (22 month)^a^	No	Yes	3 month, 10 month, 23 month
4	Conservative	No	No	No	1 week	23	Endovascular	No	No	Yes	1 week
5	Conservative	Yes (34 month)^a^	No	/	/	24	Endovascular	Lost to follow-up	/	/	/
6	Conservative	No	No	No	35 month	25	Endovascular	No	No	Yes	1 week, 12 month
7	Conservative	No	No	No	1 month	29	Endovascular	No	No	Yes	2 week
8	Conservative	No	No	/	/	31	Endovascular	No	No	Yes	1 month, 3 month
9	Conservative	No	No	No	1 week	32	Endovascular	No	No	Yes	2 week, 4 month, 12 month
10	Conservative	No	No	/	/	33	Endovascular	No	No	Yes	2 week
11	Conservative	Lost to follow-up	/	/	/	34	Endovascular	No	No	Yes	2 week
12	Conservative	Yes (38 month)^a^	No	No	3 month, 39 month	35	Endovascular	No	No	/	/
13	Conservative	No	No	No	10 month	36	Endovascular	No	No	No	2 week
14	Conservative	Lost to follow-up	/	/	/	37	Endovascular	No	No	Yes	1 week
16	Conservative	No	No	No	2 week	40	Endovascular	No	No	No	1 week
17	Conservative	Lost to follow-up	/	/	/	41	Endovascular	No	No	/	/
18	Conservative	No	No	No	1 week	44	Endovascular	No	No	Yes	4 month
19	Conservative	No	No	/	/	46	Endovascular	No	No	/	/
20	Conservative	No	No	/	/	47	Endovascular	No	No	Yes	1 month
26	Conservative	Lost to follow-up	/	/	/	49	Endovascular	No	No	Yes	1 week
27	Conservative	No	No	/	/						
28	Conservative	Lost to follow-up	/	/	/						
30	Conservative	Yes (3 month)	Yes (3 month)	No	3 month						
38	Conservative	No	No	/	/						
39	Conservative	No	No	Yes	3 month						
42	Conservative	No	No	No	2 week						
43	Conservative	Yes (1 month)	Yes (1 month)	No	1 month						
45	Conservative	No	No	Yes	4 month						
48	Conservative	No	No	/	/						
50	Conservative	No	No	/	/						
51	Conservative	No	No	/	/						

SICAD, spontaneous isolated celiac artery dissection; CTA, computed tomography angiography.

^a^ Occasional and mild relapse of symptoms.

## Discussion

SICAD is a rare condition and has not been fully investigated and reported. The experience of managing this lesion is also not comprehensively reported. Furthermore, to our knowledge, our study has the current largest sample size among published single-center studies on SICAD.

The exact etiology and risk factors of SICAD are unclear. It has been reported to occur mainly in middle-aged men (3–6, 16, 17) and is more frequently accompanied by hypertension (3, 4, 14, 16), smoking (3, 16, 17), and hyperlipidemia (4). Abdominal pain is the most common symptom of SICAD (2, 3, 11, 17). In the current cohort, the patients had similar characteristics. However, the symptoms were not specific. The incidence of misdiagnosis was reported at 13.51% in previous literature (3). In our study, three patients (5.9%) were initially misdiagnosed. For patients presenting with common symptoms, especially abdominal pain, choosing a CTA scan is a vital step in the diagnosis of symptomatic SICAD.

To date, there is no standardized treatment algorithm for symptomatic SICAD because of the heterogeneity of the clinical presentation and course of the disease (20). In general, fasting, parenteral nutrition, blood pressure control, and pain control are the more widely used approaches for conservative treatments. Antiplatelet therapy and anticoagulant therapy have been used in some patients, although their use is more controversial (7). Galastri et al. (14) used anticoagulant therapy as a routine option. In contrast, Hosaka et al. (21) suggested that anticoagulant or antiplatelet therapy was not required in most patients with this lesion. Moreover, studies have suggested that anticoagulant therapy was not required for symptomatic isolated mesenteric artery dissection without evidence of ischemia, and antiplatelet therapy or observation alone might be used (16). A systematic literature review (2) summarized the available information regarding the conservative treatment of 62 patients among 11 series; of these, 58% did not receive any specific medication for dissection, while 21%, 11.3%, and 9.7% received anticoagulants, antiplatelets, and dual therapy, respectively. Nevertheless, comorbidities of symptomatic SICAD may be complex and the use of antithrombotic agents still requires multiple considerations. The criterion for endovascular therapy has not been fully established yet as well (20). Some possible indications in previous literature could be roughly summarized as persistence or progression of symptoms despite using conservative treatment, as well as suspected visceral organ ischemia, refractory pain, compression of true lumen aneurysmal dilation (especially ≥2 cm), an extension of dissection, rupture, impending rupture, etc. (3, 6, 14, 22). Notably, in the current study, 26 (51.0%) patients met the criteria for dissecting aneurysms, but only 10 patients received endovascular treatment and only one had a diameter ≥ 2 cm. Additionally, a study on symptomatic spontaneous isolated superior mesenteric artery dissection (SISMAD) showed that nearly 70% of the cases met the diagnostic criteria of the aneurysm but the maximum diameter in almost all patients decreased significantly or remained unchanged during the mean follow-up period of 14.3 months (23).

A previous report claimed that endovascular treatment may be necessary for nearly half of the patients with symptomatic SICAD (6). In our study, a rate of 39.2% (20/51) were treated with endovascular treatment. Although results from a systematic review and meta-analysis showed that initial conservative treatment could target most patients with symptomatic SICAD and SISMAD, an estimated 8.0% and 12.0% required secondary intervention during the follow-up (7). Another study showed that 3 of 10 patients with symptomatic SICAD with initial conservative treatment underwent endovascular treatment finally due to persistent pain or progression of the dissection (14). In the current study, both exclusively conservative and interventional treatment had satisfactory outcomes. Nevertheless, two patients (7.7%) in the conservative group underwent endovascular treatment during the follow-up period, which was consistent with the systematic review (7). Encouragingly, no patients in the endovascular treatment group needed secondary endovascular intervention in this study. Previous literature demonstrated that endovascular treatment presents with better remodeling than conservative treatment (6). Moreover, endovascular treatment was reported to have favorable outcomes in terms of long-term patency and complete remodeling in both SICAD and SISMAD, especially in SICAD (9). Shi et al. (24) and Xu et al. (25) reported similar results in SISMAD. In the current study, the complete remodeling rate in the endovascular group was significantly higher than that in the conservative group (85.7%, 12/14 vs. 15.4%, 2/13; *P* < 0.001), and the process could even take only a week. Furthermore, previous literature indicated that patients treated with endovascular treatment had high persistent stability, suggesting that the approach could be reserved in case of failure of conservative treatment (3). These findings together indicate the safety, quick response, and lasting efficacy of active endovascular treatment are promising for initial treatment or to resolve a failed conservative treatment. Interestingly, of the patients with type IIB in our study, one underwent a secondary intervention and another had a relapse of symptoms. Nevertheless, none of the patients had a secondary intervention and symptomatic relapse among patients with type IB and type IIA. This suggests that type IIB may be an unstable morphological type, requiring perhaps a more aggressive endovascular treatment, while type IB and type IIA seem to present as more benign. However, the level of evidence is still limited by the sample size, study type, and follow-up time. A further randomized controlled trial with long-term follow-up is needed.

A previous study (3) indicated the length of dissection was a risk factor for conservative treatment failure, together with branches’ involvement. We found that the length of dissection was significantly larger in the endovascular group than in the conservative group (25.5 ± 11.8 mm vs. 19.1 ± 7.4 mm, *P* = 0.022), which may indicate that the length of the dissection might have potential relevance to the selection for endovascular treatment for symptomatic SICAD. This seems intuitive as longer lesions might indicate a greater impact on the disease condition. However, we did not find a difference in the extent of branches’ involvement between the two groups. Some researchers believe that the extent of branches’ involvement might not affect the treatment strategy and outcomes of SICAD (1).

The current study has some limitations. First, this was a retrospective study with its inherent limitations, such as selection bias. Second, the sample size was relatively small and there were some losses during follow-up, which might affect the interpretation ability of the results. Therefore, further studies with larger sample sizes and longer follow-up periods are necessary. Third, few CTA scans were obtained, and the estimated complete remodeling rate might be biased; however, the advantage of endovascular treatment in remodeling was clear, and the follow-up is ongoing. Fourth, the study could not determine whether the medical treatment was homogeneous and optimal in each patient in the conservative group. Finally, data were obtained from a single center, which may limit the generalization of the findings.

## Conclusion

In the current study, patients with symptomatic SICAD who were selectively treated with conservative treatment or endovascular treatment were found to show satisfactory outcomes in the early and medium follow-up periods. Endovascular treatment showed significant advantages with regard to complete remodeling of the celiac artery and presented with a lower rate of secondary intervention. Furthermore, it is suggested to be a safe and effective remedy for failed conservative treatment.

## Data Availability

The raw data supporting the conclusions of this article will be made available by the authors, without undue reservation.
